# Comparing Health-Related Quality of Life of Cancer Patients under Chemotherapy and of Their Caregivers

**DOI:** 10.1100/2012/135283

**Published:** 2012-04-24

**Authors:** Ioannis Vrettos, Konstantinos Kamposioras, Nick Kontodimopoulos, Evelina Pappa, Elissavet Georgiadou, Dionysios Haritos, Angelos A. Papadopoulos, Dimitris Niakas

**Affiliations:** ^1^Second Department of Internal Medicine, Medical School, Attikon University General Hospital, P.C. 12462 Athens, Greece; ^2^Faculty of Social Sciences, Hellenic Open University, P.C. 26222 Patras, Greece; ^3^Department of Pathology, A. Fleming General Hospital, P.C. 15127 Athens, Greece

## Abstract

*Introduction*. Cancer is a major disorder physically and psychologically affecting both patients and their caregivers. In this study, health-related quality of life (HRQoL) of patient-caregiver dyads during the period of chemotherapy was assessed. *Material and Methods*. Two hundred twenty-two cancer patient-caregiver dyads were enrolled in the study, which was conducted from October 2008 to March 2009. HRQoL was evaluated with EQ-5D. *Results*. The mean age of the sample was 57.4 and 48.9 for patients and caregivers, respectively. The EQ-5D descriptive system indicates that female patients more frequently experience anxiety and depression than male patients. Male and higher-education caregivers had higher VAS scores, while demographic factors did not seem to influence patients' HRQoL. Anxiety and depression of caregivers were correlated with patients' problems in self-care and usual activities. *Conclusions*. Quality of life is highly influenced during the period of chemotherapy for both patients and caregivers and is often under reported. Interventions that can improve HRQoL, especially in the domain of mental health for both cancer patients and their caregivers, need to be implemented.

## 1. Introduction

Cancer is one of the five leading causes of death in all age groups in both males and females in the USA. Moreover, it is the main cause of death among women of ages 40 to 79 years and among men of ages 60 to 79 years. Since there has been a notable improvement in the relative 5-year survival rates for many cancer types and for all cancers combined [1], an increasing interest for the impact of the disease on quality of life of cancer patients has emerged [2–4].

Moreover, cancer is a major disorder which affects not only the patients themselves, but their family and relatives as well. Previous research has demonstrated that caring for patients with cancer has a considerable impact on the caregiver [5–10]. It has been reported that being a caregiver for a patient with cancer is associated with anxiety [5], depression [6], sleep disturbance [7], fatigue [8], impaired quality of life [9], impact on work, and economic burden [10].

In order to assess the health-related quality of life (HRQoL) of cancer patients, both generic and disease-specific questionnaires have been applied [11]. Although disease-specific questionnaires appeared to be more responsive than generic instruments [12], there is evidence that the sensitivity of the generic EQ-5D questionnaire is comparable with the disease specific EORTC QLQ C-30 [13]. On the other hand, generic questionnaires have wide applicability across conditions and interventions. They can also be used to compare different cancer patient groups, cancer patients and the general population or other diverse populations [12]. Among the generic questionnaires available, the EQ-5D is a widely used HRQoL instrument that has only five items and it is easy to administer and complete [14].

The EQ-5D has been increasingly used recently in cancer patients, and the growing body of literature provides evidence to support its validity and reliability [15]. It has been used mainly to study cancer patient groups with the same primary tumor site [16–18] and, occasionally, irrespectively of the primary site [15, 19, 20].

The purpose of our survey was to investigate the HRQoL of cancer patients and their caregivers during the period of chemotherapy and to assess the impact of various demographic parameters on the quality of life of the dyad, using the EQ-5D.

## 2. Materials and Methods

### 2.1. Study Population

From October 2008 to March 2009, two hundred twenty-two cancer patients attending the oncology day clinic of our hospital and their accompanying person (hereinafter designated as the “caregiver”) were enrolled in the study. Two dedicated investigators conducted the interview-based cross-sectional survey of the target population.

Eligibility criteria for the patients and their caregivers included being more than 18 years old and being physically and mentally well to communicate with the interviewers. Moreover, eligible patients were those with an Eastern Cooperative Oncology Group (ECOG) performance status between 0 and 2 [21] and being on active treatment for either adjuvant or palliative intent. The patients and their caregivers provided informed consent to participate in the study.

Patients' demographic and social characteristics (gender, age, marital status, and educational level), disease primary site, and comorbidities (hypertension, coronary heart disease, diabetes mellitus, and other) were also recorded by the interviewers. Data relating to the specific patient-caregiver relationship were also recorded (i.e., spouse, parent, or offspring, if he/she was the main caregiver and if they lived together).

The study was approved by the ethical and scientific committee of Attikon University Hospital, Athens, Greece.

### 2.2. Instrument

The EQ-5D [22] is a short, generic, HRQoL instrument that consists of the EQ-5D descriptive system, the EQ visual analogue scale (EQ-VAS), and the EQ-5D utility index. The descriptive system assesses five dimensions: mobility, self-care, usual activities, pain/discomfort, and anxiety/depression. Each dimension is subdivided in three levels of severity (no complaints, some complaints, severe complaints), and the respondent is asked to indicate the most appropriate answer for her/his health state. This decision results in a 1-digit number expressing the level selected for that dimension. A combination of these answers defines the respondent's health-state expressed as a 5-digit health status profile. Totally, 243 (3^5^) possible health status profiles are defined. On the EQ-VAS, respondents are asked to rate their overall health state between 0 and 100 on a 20 cm vertical visual analogue scale, were 0 is the worst imaginable health state, and 100 is the best imaginable health state. The EQ-5D index is derived from time trade-off valuations from a general UK population [23, 24].

The EQ-5D was found to be applicable and adaptable to the Greek environment [25], and its construct validity was demonstrated [26].

### 2.3. Statistical Analysis

Nonparametric tests (Mann-Whitney and Kruskal-Wallis) were used to assess the socioeconomic and clinical differences in EQ-5D VAS and Index Scores in both patient and caregivers subgroups. The chi-square test was applied to evaluate the differences in response frequencies between the five dimensions of EQ-5D in patients and their caregivers as well as to assess the gender differences in the five EQ-5D dimensions. Spearman test was used to find out relationships between patients and caregivers HRQoL dimensions of EQ-5D. Results were considered statistically significant when *P* < 0.05, and all analyses were performed using SPSS v16.0.

## 3. Results

Of the 222 eligible patient-caregiver dyads, 212 finally participated in the study (response rate 96.5%). From the rest of the cases, six relatives and four patients were reluctant to participate. Patients' and caregivers' sociodemographic and clinical characteristics, as well as the mean EQ-5D VAS and Index Scores, are presented in Tables 1 and 2, respectively. The mean age of the study participants was 57.4 years ±14.6 (M ± SD) for the patients and 48.9 years ±14.3 for the caregivers, and the majority was females (56.1% and 62.7%, resp.). 170 patients (80.2%) and 160 caregivers (75.5%) were married, while 120 patients (56%) and 168 caregivers (79.2%) had secondary and higher education, respectively. Gastrointestinal (26.9%) and urogenital (21.7%) malignancies were the most prevalent cancer types followed by breast (18.4%), respiratory (15.6%), and head/neck cancers (5.7%). Caregivers lived in the same house and were the “main caregiver” in 73.1% of the cases, whereas in 46% of the cases was a spouse.

Distribution of EQ-5D dimensions (mobility, self-care, usual activities, pain/discomfort, and anxiety/depression) of the patients and the caregivers are summarized in Table 3. Comparing the HRQoL of male and female patients, we observed that female patients were more likely to have anxiety/depression (*χ* ^2^ = 17.4, *P* < 0.001), whereas, for caregivers, we found that female caregivers' HRQoL was worse in the dimensions of mobility (*χ* ^2^ = 3.98, *P* = 0.046), pain/discomfort (*χ* ^2^ = 14.96, *P* = 0.001), and anxiety/depression (*χ* ^2^ = 8.78, *P* = 0.012) (Table 4).

The correlation between patients' and caregivers' HRQL indicated that problems in patients' dimensions of self-care and usual activities had a negative influence in the dimension of anxiety/depression of the caregivers (*P* = 0.039 and *P* = 0.033, resp.).

The most prevalent health profiles, out of a total of 243 possible health states for the patients and their caregivers, are described in Table 5. The most frequent health state of both patients and caregivers was 11112 that indicates moderate anxiety or depression and no problems in the other dimensions (14.6% and 48.1%, resp.), followed by the health state 11111 that indicates no problems in any of the five dimensions (9.9% and 16.5%, resp.).

## 4. Discussion

It is well documented [27–29] that HRQoL in general population, as it is measured with the EQ-5D, is influenced by sociodemographic differences like gender, educational level, and marital status. Fewer problems on the descriptive system and higher scores on the visual analogue scale are most prevalent in males, higher-educational-level groups, and married people.

 In our study, according to the subjective state of health recorded on the visual analogue scale, male and higher-education caregivers had higher VAS scores. Interestingly, married caregivers had lower VAS scores than single ones in contrast with the general population's observations. This can be explained by the fact that the higher proportion of caregivers in the current study was spouses of the patients with whom they were living with in the same house and caring for. According to Nijboer et al. [30], being a partner of a care recipient, as compared to other caregivers, is associated with experiencing more strain, potentially becoming ill, and experiencing higher levels of psychiatric symptoms.

On the contrary, these monitored factors (gender, marital status, and educational level) had no influence on the subjective health condition of the patients, as recorded by the EQ-VAS scores in the present study. Similarly, Slovacek et al. [31] reported that the influence of these factors on EQ-5D VAS, in patients who have undergone hematopoietic stem cell transplantation, was not proven to be statistically significant. Another study of men with prostate cancer has reported the absence of a significant association between marital status and the EQ-5D VAS [32].

The EQ-5D item responses indicate that female patients experience more frequently anxiety or depression than male patients. Previous studies have demonstrated high levels of depression and anxiety in cancer patients. No gender differences for anxiety [33, 34] and depression [34, 35], a higher prevalence of anxiety [35–38] or depression [38, 39] in women or a higher prevalence of depression in men [40], have been reported in various studies. On the other hand, HRQoL of female caregivers was worse than that of males in all five dimensions of the EQ-5D descriptive system and this was shown to be statistically significant for three dimensions. Caregiving is physically and emotionally demanding, and gender differences, in self-reported caregivers' physical and psychological health, have been widely reported in previous studies [9, 41–43].

Cancer patients and their caregivers had statistically significant differences in all but one EQ-5D dimension (Table 3). Despite that a higher proportion of caregivers experienced moderate or extreme anxiety or depression (80.2%) than patients did (74%), this was not statistically significant. Likewise, previous studies have noted higher rates of psychological problems in caregivers as compared with patients. In a study of gastrointestinal and lung cancer patients [44], symptoms of depression were reported in 38.9% of the caregivers and in 23% of their ill spouses. In another study of patients with brain tumors [45], the spouses of the patients were more psychologically affected than the patients (47% and 38%, resp.). Moreover, Bambauer et al. [46] demonstrated that the presence of anxiety disorders in one member of the dyad increased the likelihood of an anxiety disorder to the partner. Similarly, Fleming et al. [47] have reported a correlation between advanced metastatic cancer patients' and caregivers' mental health and depression scores.

As expected, caregivers' anxiety and depression were highly correlated with patients' problems in self-care and usual activities. These findings support previous study findings that caregivers' depression and patients' disability to perform their daily activities are highly related. Emanuel et al. have found that caregivers of terminally ill patients who needed a high amount of assistance (transportation, nursing care, homemaking, and personal care) were significantly more likely to have depressive symptoms than caregivers of patients with low care needs [48]. Similarly, patients' dependency in activities of daily living correlated with caregivers' depression symptoms [49]. On the other hand, Haley et al. in a study of hospice patients with lung cancer or dementia found that objective measures of patient impairment or amount of care provided are not strong predictors of caregiver depression [50]. Moreover, caregivers who subjectively appraised caregiving tasks as less stressful had lower depression. It is worth noting that patients' pain was not related with caregivers' anxiety and depression. Previous studies have demonstrated that patients' symptoms were a significant predictor of caregiver depression [51] or that those caregivers of patients with cancer-related pain scored higher for depression than caregivers of patients without cancer-related pain [6].

The difference observed between EQ-5D index and VAS, especially in marital status, education, comorbidity, and the type of caregiver (Table 2), has been already observed in comparative studies concerning psychiatric disorders [52, 53]. Although both items measure quality of life, it seems that EQ-5D index is less responsive and need larger patient samples to detect meaningful differences compared with EQ VAS. Nevertheless, both items seem to have equal validity in prostate cancer patients but these results have to be validated in further larger studies with cancer patients [54].

We are aware of the fact that our study can be limited by some factors. First, there is a limitation associated with the EQ-5D itself. Health-related quality of life measurement in cancer patients is usually assessed using cancer-specific instruments that are likely to be more responsive than generic instruments [12]. However, in this case, a disease-specific instrument would not allow us to make a comparison between two different populations, like cancer patients and their relatives. Secondly, study participants were patients and their relatives that accompanied them on the day of chemotherapy in the oncology day clinic and not always the main caregiver nor the person who lived in the same house, so study findings cannot be generalized to all cancer patient-caregiver dyads.

Despite the limitations, the present study represents an attempt to understand the complicated interaction between cancer patients undergoing chemotherapy and their relatives, in terms of their health-related quality of life. In modern medicine, the evaluation of a patient's health problem is based not only on clinical or laboratory markers but also on a holistic approach of the patient that includes the evaluation of the consequences of diagnosis or therapy of the health condition. Interventions that can improve HRQoL, especially in the domain of mental health, of both cancer patients and their caregivers need to be implemented.

## 5. Conclusions

- Both cancer patients and caregivers were highly affected psychologically of the disease.
- Demographic characteristics that influence the subjective health status of caregivers did not appear to influence the subjective health status of the patients.
- Female patients appear to be more anxious or depressed than males.
- Anxiety and depression of caregivers were correlated with patients' problems in self-care and usual activities.

## Figures and Tables

**Table 1:** Patients' sociodemographic and clinical characteristics and EQ-5D VAS and Index Scores.

	*N* (%)	VAS ± 1SD	EQ-5D_index_ ± 1SD
Gender			
Females	119 (56.1)	64.7 ± 22.7	0.612 ± 0.341
Males	93 (43.9)	68.9 ± 20.4	0.707 ± 0.318
P*		0.268	0.017

Age			
Mean	57.4		
Range	18–81		
Marital status			
Married	170 (80.2)	66.6 ± 22.4	0.637 ± 0.350
Unmarried	19 (9.0)	65.7 ± 21.1	0.718 ± 0.252
Divorced	5 (2.6)	57.0 ± 19.2	0.763 ± 0.182
Widowed	14 (6.6)	68.6 ± 17.4	0.683 ± 0.292
P**		0.686	0.936

Education			
Primary	77 (36.3)	68.4 ± 21.7	0.612 ± 0.360
Secondary	74 (34.9)	65.1 ± 21.7	0.659 ± 0.336
Technological education institution	18 (9.1)	69.7 ± 23.9	0.666 ± 0.338
University	28 (14.2)	63.7 ± 18.9	0.750 ± 0.219
P**		0.664	0.579

Comorbidity			
Hypertension			
Yes	63 (29.7)	68.9 ± 21.2	0.62 ± 0.34
No	149 (69.3)	65.6 ± 22.11	0.66 ± 0.33
P*		0.443	0.468
Coronary Heart disease			
Yes	27 (12.7)	73.3 ± 19.0	0.61 ± 0.35
No	185 (86.3)	65.6 ± 22.1	0.65 ± 0.33
P*		0.151	0.775
Diabetes mellitus			
Yes	22 (10.4)	74.3 ± 20.9	0.58 ± 0.32
No	190 (89.6)	65.7 ± 21.8	0.66 ± 0.33
P*		0.088	0.145

Cancer site			
Gastrointestinal	57 (26.9)	66.3 ± 22.5	0.60 ± 0.38
Urogenital	46 (21.7)	65.9 ± 22.6	0.61 ± 0.29
Breast	39 (18.4)	66.3 ± 20.3	0.69 ± 0.29
Respiratory	33 (15.6)	68.2 ± 22.7	0.63 ± 0.40
Head and neck	12 (5.7)	70.4 ± 19.1	0.85 ± 0.13
Other	25 (11.8)	65.1 ± 22.3	0.68 ± 0.29
P**		0.861	0.166

*Mann-Whitney; **Kruskal Wallis.

**Table 2:** Caregivers' sociodemographic and clinical characteristics, EQ-5D VAS, and Index Scores.

	*N* (%)	VAS ± 1SD	EQ-5D_index_ ± 1SD
Gender			
Females	133 (62.7)	69.6 ± 21.8	0.783 ± 0.228
Males	79 (37.3)	75.9 ± 14.9	0.895 ± 0.141
P*		0.023	<0.001

Age			
Mean	48.9		
Range	20–80		
Marital status			
Married	160 (75.5)	71.17 ± 18.7	0.831 ± 0.201
Unmarried	36 (17.0)	79.4 ± 18.6	0.865 ± 0.173
Divorced	8 (3.8)	70.7 ± 19.2	0.750 ± 0.256
Widowed	6 (2.8)	55.8 ± 26.2	0.695 ± 0.267
P**		0.018	0.177

Education			
Primary	30 (14.2)	66.3 ± 19.5	0.872 ± 0.166
Secondary	91 (42.9)	71.2 ± 20.1	0.813 ± 0.209
Technological educational institution	33 (16.7)	73.3 ± 16.6	0.851 ± 0.169
University	44 (22.3)	80.1 ± 11.9	0.827 ± 0.209
P**		0.009	0.493

Comorbidity			
Hypertension			
Yes	37 (17.5)	67.43 ± 23.44	0.83 ± 0.22
No	175 (82.5)	74.94 ± 18.74	0.82 ± 0.20
P*		0.206	0.588
Coronary Heart disease			
Yes	14 (6.6)	61.07 ± 28.22	0.78 ± 0.27
No	198 (93.4)	72.94 ± 18.74	0.82 ± 0.20
P*		0.141	0.481
Diabetes mellitus			
Yes	11 (5.2)	57.27 ± 27.32	0.80 ± 0.30
No	201 (94.8)	72.78 ± 18.96	0.82 ± 0.20
P*		0.048	0.570

Relationship			
Spouse	98 (46.2)	71.4 ± 19.4	0.84 ± 0.19
Parent	40 (18.9)	71.4 ± 24.8	0.80 ± 0.23
Offspring	47 (22.2)	76.5 ± 15.3	0.85 ± 0.16
Other	27 (12.7)	67.0 ± 18.6	0.75 ± 0.25
P**		0.183	0.367

Main caregiver			
Yes	155 (73.1)	70.5 ± 20.1	0.82 ± 0.21
No	57 (26.9)	79.6 ± 16.0	0.84 ± 0.18
P*		0.009	0.686

Living in the same house			
Yes	155 (73.1)	70.6 ± 20.6	0.82 ± 0.20
No	57 (26.9)	75.6 ± 16.6	0.82 ± 0.21
P*		0.113	0.800

*Mann-Whitney; **Kruskal Wallis.

**Table 3:** Patients' and caregivers' EQ-5D dimension scores.

	Patients	Caregivers
	*N* (%)	*N* (%)
Mobility		
No difficulties	137 (64.6)	201 (94.8)
Some difficulties	68 (32.1)	11 (5.2)
Extreme difficulties	7 (3.3)	0 (0)
*P*	*χ* ^2^ = 60.24; df = 2; *P* < 0.001	
Self-care		
No difficulties	171 (80.7)	210 (99.1)
Some difficulties	34 (16.0)	2 (0.9)
Extreme difficulties	7 (3.3)	0 (0)
*P*	*χ* ^2^ = 39.43; df = 2; *P* < 0.001	
Usual activities		
No difficulties	105 (49.5)	196 (92.5)
Some difficulties	89 (42.0)	16 (7.5)
Extreme difficulties	18 (8.5)	0 (0)
*P*	*χ* ^2^ = 96.26; df = 2; *P* < 0.001	
Pain/discomfort		
No difficulties	99 (46.7)	173 (81.6)
Some difficulties	88 (41.5)	38 (17.9)
Extreme difficulties	25 (11.8)	1 (0.5)
*P*	*χ* ^2^ = 62.13; df = 2; *P* < 0.001	
Anxiety/depression		
No difficulties	55 (25.9)	42 (19.8)
Some difficulties	122 (57.5)	127 (59.9)
Extreme difficulties	35 (16.5)	43 (20.3)
*P*	*χ* ^2^ = 2.76; df = 2; *P* = 0.259	

df: degrees of freedom.

**Table 4:** Intragender differences in patients' and caregivers' EQ-5D dimensions.

	Patients		Caregivers	
	Males	Females	Males	Females
	*N* (%)	*N* (%)	*N* (%)	*N* (%)
Mobility				
No difficulties	63 (67.7)	74 (62.2)	78 (98.7)	123 (92.5)
Some difficulties	29 (31.2)	39 (32.8)	1 (1.3)	10 (7.5)
Extreme difficulties	1 (1.1)	6 (5.0)	0 (0)	0 (0)
	*χ* ^2^ = 2.78; df = 2; *P* = NS		*χ* ^2^ = 3.98; df = 2; *P* = 0.046	
Self-care				
No difficulties	77 (82.8)	94 (79.0)	79 (100)	131 (98.5)
Some difficulties	12 (12.9)	22 (16.5)	0 (0)	2 (1.5)
Extreme difficulties	4 (4.3)	3 (2.5)	0 (0)	0 (0)
	*χ* ^2^ = 1.61; df = 2; *P* = NS		*χ* ^2^ = 1.21; df = 1; *P* = NS	
Usual activities				
No difficulties	47 (50.5)	58 (48.7)	76 (96.2)	120 (90.2)
Some difficulties	41 (44.1)	48 (40.3)	3 (3.8)	13 (9.8)
Extreme difficulties	5 (5.4)	13 (10.9)	0 (0)	0 (0)
	*χ* ^2^ = 2; df = 2; *P* = NS		*χ* ^2^ = 2.58; df = 1; *P* = NS	
Pain/discomfort				
No difficulties	45 (48.4)	54 (45.8)	75 (94.9)	98 (73.7)
Some difficulties	35 (37.6)	53 (44.5)	4 (5.1)	34 (25.6)
Extreme difficulties	13 (14.0)	12 (10.1)	0 (0)	1 (0.8)
	*χ* ^2^ = 1.37; df = 2; *P* = NS		*χ* ^2^ = 14.96; df = 2; *P* = 0.001	
Anxiety/depression				
No difficulties	34 (36.6)	21 (17.6)	20 (25.3)	22 (16.5)
Some difficulties	53 (57.0)	69 (58.0)	51 (64.6)	76 (57.1)
Extreme difficulties	6 (6.5)	29 (24.4)	8 (10.1)	35 (26.3)
	*χ* ^2^ = 17.4; df = 2; *P* < 0.001		*χ* ^2^ = 8.78; df = 2; *P* = 0.012	

df: degrees of freedom.

**Table 5:** The most prevalent health profiles of the patients and their caregivers.

Patients' health state	Frequency (%)	Caregivers' health state	Frequency (%)
11112	31 (14.6)	11112	102 (48.1)
11111	21 (9.9)	11111	35 (16.5)
11122	21 (9.9)	11113	29 (13.7)
11212	11 (5.2)	11122	12 (5.7)
21222	10 (4.7)	11121	6 (2.8)
11113	8 (3.8)	11123	6 (2.8)
11121	8 (3.8)	11212	4 (1.9)
21212	8 (3.8)	21223	3 (1.4)
